# Supplementary material for: The global antigenic diversity of swine influenza A viruses
Source: eLife. 2016 Apr 22;5:e12217. doi: 10.7554/eLife.12217 (PMC4846380; doi:10.7554/eLife.12217)
Supplement: Supplementary file 3. — DOI: http://dx.doi.org/10.7554/eLife.12217.014 [file elife-12217-supp3.docx]

Supplementary File 3_Table3

Overall drift rate in antigenic units per year for H1 and H3 swine influenza virus lineages and 95% credible interval (HPD)

| **Lineage** | **Antigenic drift rate per year (AU)** | **Mean pairwise distance**  **(95% HPD)^a^** |
| --- | --- | --- |
| **H1** |  |  |
| Classical swine 1A | 0.15 | 0.10 – 0.20 |
| Human seasonal-like swine 1B |  |  |
| *European introduction* | 0.17 | 0.04 – 0.31 |
| *USA Delta-1 introduction* | 0.63 | 0.27 – 1.00 |
| *USA Delta-2 introduction* | 0.85 | 0.04 – 1.68 |
| Eurasian avian-like swine 1C | 0.15 | 0.03 – 0.26 |
|  |  |  |
| **H3** |  |  |
| European swine 3A | 0.28 | 0.10 – 0.45 |
| USA swine 3B | 0.49 | 0.12 – 0.83 |
