# Supplementary material for: The global antigenic diversity of swine influenza A viruses
Source: eLife. 2016 Apr 22;5:e12217. doi: 10.7554/eLife.12217 (PMC4846380; doi:10.7554/eLife.12217)
Supplement: Supplementary file 4. — DOI: http://dx.doi.org/10.7554/eLife.12217.015 [file elife-12217-supp4.docx]

Supplementary Table4. Summary of previously reported rates of antigenic drift (in antigenic units per year) of influenza A viruses.

| **Lineage** | **Timespan studied** | **Antigenic drift rate (antigenic units per year)** | **Source** |
| --- | --- | --- | --- |
| **H1** |  |  |  |
| Human H1 | 1977 – 2009 | 0.62 (95% HPD: 0.56 – 0.67) | Bedford et al. 2014 |
|  |  |  |  |
| **H3** |  |  |  |
| Human H3 | 1982 – 2002 | 2.0 (R^2^ = 0.86) | de Jong et al. 2007 |
|  | 2002 – 2007 | 2.13 | Russell et al. 2008 |
|  | 1968 – 2011 | 1.01 (95% HPD: 0.98 – 1.04) | Bedford et al. 2014 |
| European swine 3A | 1984 – 1999 | 0.3 (R^2^ = 0.64) | de Jong et al. 2007 |
